# Facing Gold Crowns with Porcelain

**Published:** 1900-04-15

**Authors:** George Evans


### FACING GOLD CROWNS WITH PORCELAIN.

BY GEORGE EVANS, D.D S.

Gold crowns can be enameled with the Jenkins porcelain very artistically. A seamless crown is shaped to the exact form of the tooth in this way: Form a little tube of copper that fits the tooth, without any solder, and take an impression of the tooth in plaster. Take the tube from the tooth, wind around it a piece of paper, pour in fusible metal and form a die. Lengthen slightly the shank of the die on its removal from the tube. If desirable, pour another one in the tube to use as a guide in shaping the neck of the gold crown, so as to very nearly meet the margin of the gum on its being adjusted in the mouth. Then shape a stamped-up, ready-made seamless crown on the first die by driving the die into the crown, using as a matrix a folded napkin or towel. Thus in a few minutes the gold crown is shaped to the exact form of the tooth. The crown must either be seamless, or soldered with pure gold. The gold of which the crown is composed will have to be alloyed with platinum. This permits it being soldered with pure gold. The crown being adjusted on the tooth, mark on the labial surface the amount of gold that is exposed on viewing the patient directly from the front. Then remove the gold crown and thin the marked labial surface enough to admit of the gold of the crown being slit close to the grinding-surface, and pressed inward against the tooth to give room for the porcelain body that is to be placed on the crown. Then perforate this compressed section of the gold, and fuse the Jenkins body on the gold. It requires about four bakings to perform the operation. If it is a pulpless tooth, you can take much more space for the presence of the porcelain body, by removing what is necessary of the natural tooth. Where there is a badly broken-down tooth, simply a root, I insert posts in the root and shape down the coronal section with amalgam. This specimen has an S. S. White tooth ground down to a mere wafer fastened in the front of the gold crown with the Jenkins porcelain body. The process of inlaying crowns with porcelain is specially intended for teeth with living pulps. The one I baked this afternoon has the porcelain anchored inside the crown, in a mass down inside the grinding-surface, and is anchored there in such a manner that it is impossible for it to break or loosen at that point, which is where the force of the occlusion comes. The part that extends up over the labial portion does not receive much pressure. The oxyphosphate, being behind the gold, stiffens and supports both gold and porcelain. I soldered in mere films of porcelain on crowns and cemented the crowns on teeth years ago, and they are there yet. Some of them have checked, but I have not seen any come off.

DISCUSSION.

Dr. A. L. Northrop: If you use this porcelain in cases where there is strain, will it loosen or crack the porcelain in the crown?

Dr. Evans: Yes, it is possible. I have only recently gone into this particular style of porcelain work. I present this subject to you at the request of your committee. As to the practical result, I do not want to place myself on record as saying that this operation is a reliable one, but, from the way it looks, I think it will enable us in many cases to cover over and hide the glaring gold that is so often displayed in crowns in the front of the mouth.

The gold crowns that are enameled have been first reinforced with pure gold. One way is to clean perfectly the inside grinding-surface of a gold crown and scratch the surface, and with a sharp plugger take some freshly annealed pellets of foil and pack them in just where you want to thicken it. You pack the gold the same as a filling. The gold adheres. You can then take a small-pointed agate burnisher and burnish it solid. You thus instantly form solid cusps in a gold crown. I also put a piece of pure gold with a little flux on it in one of these gold crowns and melt it into the cusps. I also slit a crown and drew it in and soldered it with pure gold, 24 k. There is a point to be explained in this. Some gentlemen look with apprehension when they see me do this. You should not heat up the gold plate quickly or hotter than is necessary to just melt your solder. If your solder is not heated in proportion with the crown, the moment it melts it perforates the overheated gold, and that is the way a hole is melted in the crown. Put in a piece of 18 k. gold or even 14, and hold it over the flame of a Bunsen burner; heat it suddenly, and you will see the crown get to a pink shade with the solder still dark. The moment the solder does melt, the crown being hotter than the solder, it has such an affinity for the gold, the solder goes through the crown. When I put in pure gold and gradually heat it up you can see that both crown and solder are the same temperature, the pure gold melts at a slightly lower temperature than the crown, and, when it fuses, flows on the surface of the crown. The crown is made of 23 k. gold, alloyed with platinum. Pure gold can, therefore, be melted on it.

Dr. Littig: After you used a method by which you compressed the outside of the gold, perforated it, and baked the porcelain on the outside, do you find this as strong?

Dr. Evans: That was done with the glass filling, and I found it discolored in time, and I had to go over the operation. This process I described to-day is in some respects similar. In the present method, the heat required for the Jenkins porcelain is about 100° to 150° below the melting point of pure gold. I accordingly use pure gold to do the soldering.

Dr. Littig: Would not copper in the solder discolor the porcelain?

Dr. Evans: Yes; that is the reason I use pure gold. All porcelains are very sensitive to any of the base metals, and you can not put any base alloy in gold used in connection with the fusing of any porcelain that I know of. I call the Jenkins body a porcelain, fusing at a low temperature.—*Dental Cosmos, page* 179, 1900.

